# Production, purification and characterization of halophilic organic solvent tolerant protease from marine crustacean shell wastes and its efficacy on deproteinization

**DOI:** 10.1007/s13205-016-0474-y

**Published:** 2016-07-26

**Authors:** Thirumalai Maruthiah, Beena Somanath, Jebamonydhas Vijila Jasmin, Grasian Immanuel, Arunachalam Palavesam

**Affiliations:** 1 Centre for Marine Science and Technology, Manonmaniam Sundaranar University, Rajakkamangalam, Kanyakumari, 629 502 Tamilnadu India; 2 Department of Zoology, Rani Anna Government College for Women, Manonmaniam Sundaranar University, Tirunelveli, 627 012 Tamilnadu India; 3 Department of Zoology, Muslim Arts College, Thiruvithancode, Kanyakumari, 629 174 Tamilnadu India; 4 Department of Animal Science, Manonmaniam Sundaranar University, Tirunelveli, 627 012 Tamilnadu India

**Keywords:** Halophilic organic solvent tolerant protease, Marine crustacean shell wastes, Stain removal, Deproteinization, Antioxidants

## Abstract

The quantum of marine fish wastes produced by fish processing industries has necessitated to search new methods for its disposal. Hence, this study is focused on production and purification of halophilic organic solvent tolerant protease (HOSP) from marine *Alcaligenes faecalis* APCMST-MKW6 using marine shell wastes as substrate. The candidate bacterium was isolated from the marine sediment of Manakudi coast and identified as *A. faecalis* APCMST-MKW6. The purified protease showed 16.39-fold purity, 70.34 U/mg specific activity with 21.67 % yield. The molecular weight of the purified alkaline protease was 49 kDa. This purified protease registered maximum activity at pH 9 and it was stable between pH 8–9 after 1.30 h of incubation. The optimum temperature registered was 60 °C and it was stable between 50 and 60 °C even after 1.30 h of incubation. This enzyme also showed maximum activity at 20 % NaCl concentration. Further, manganese chloride, magnesium chloride, calcium chloride and barium chloride influenced this enzyme activity remarkably and it was also found to be enhanced by many of the tested surfactants and solvents. The candidate bacterium effectively deproteinized the shrimp shell waste compared to the other tested crustaceans shell wastes and also attained maximum antioxidant activity.

## Introduction

Halophilic organic solvent tolerant bacteria are one of the most important groups involved in the extremophiles field. Among this, bacterial proteins from the relevant bacteria tolerate well in different concentrations of salts and organic solvents due to their extreme characteristics. As of now halophilic tolerant proteins are received much attention in industrial sectors due to their applied extreme potentials. The specific advantages of the halophilic organic solvent tolerant proteases are; cell enlargement, strengthening of cell membrane, degradation and biotransformation of organic solvents and solvent efflux pump (Rahman et al. 2006). Also proteases that can mediate catalysis in non aqueous solvents tender new possibilities, such as shifting of thermodynamic balance in favor of synthesis, improving the solubility of hydrophobic substrates and products, facilitating the product recovery, and improving the thermal stability of the enzymes (Doukyu and Ogino 2010). Among the microbial proteases, alkaline proteases occupy 60 % of the total world enzyme production, with specific applications in bakery, brewing, detergent, diagnostic reagents, feeds modification, leather finishing, laundry additives, pharmaceuticals, and peptide synthesis, silk, silver recovery from X-ray/photographic film, soy processing, and waste treatment (Rajkumar et al. 2011).

Marine industry generates 50–60 % of the total weight of shellfish as waste consists of protein (35–50 %), chitin (15–25 % of dry weight) and inorganic compound (calcium carbonate) which are considered as major environmental pollutants due to uncontrolled dumping (Islam et al. 2004; Sachindra et al. 2005). Bioconversion of these materials has been proposed as waste treatment alternate to the disposal of shellfish wastes. The utilization of these wastes not only solves the environmental problem but also decreases the production costs of microbial proteases. Halophilic solvent-tolerant bacteria make up a group of microorganisms are becoming more popular in academia and industry, due to their unique ability to live in presence of organic solvents. Some of these bacteria have been found to produce haloalkaline as well as solvent-tolerant enzymes, but only a few reports are available regarding the purification and characterization of the halophilic organic solvent-tolerant protease (Annamalai et al. 2012, 2014a, b; Maruthiah et al. 2014, 2015).

The bioconversion of marine shell wastes by various marine coastal proteolytic bacterial species was reported by several authors (Annamalai et al. 2012, 2014a, b; Maruthiah et al. 2014, 2015). However, the application of *Bacillus* sp. for the deproteinization of marine crustacean wastes is rarely seen. In this study, the protease-producing *Bacillus* sp. can be used for deproteinization of crustacean wastes. Generally, the preparation of chitin from various crustacean shells involves demineralization and deproteinization with the use of strong acids or bases. Typically to overcome the scarcity of the chemical treatments, microorganisms and proteolytic enzymes were effectively used to deproteinze the crustacean wastes (Jellouli et al. 2011; Haddar et al. 2011; Ghorbel-Bellaaj et al. 2012; Maruthiah et al. 2015; Hajji et al. 2015). Considering the above facts discussed in this study, an attempt has been made on the production and purification of the HOSP from the coastal sedimentary bacterium using marine shell wastes. Further, the candidate strain also performed maximum deproteinization and antioxidant efficiency using shrimp shell waste.

## Materials and methods

### Marine fish waste powder preparation for HOSP production

The shrimp shell powder (SSP), crab shell powder (CSP) and lobster shell (LSP) used in this study were prepared from the respective shell wastes collected from local fish processing units. After collection in aseptic condition, the shell wastes were washed thoroughly with tap water and sun dried. The dried materials were milled and sieved (100 µM) to get uniform fine powder and used as sole carbon sources for protease production.

### Isolation of HOSP bacterium and culture conditions for protease production

The candidate bacterium was isolated from the sediment sample of Manakudi coast (Lat. 8°05N: Lon. 77°32′E), Kanyakumari District, India and it was identified based on the morphological, physiological, and biochemical characteristics as well as 16S rRNA sequencing. The isolation and identification of potent HOSP strain was followed using solvent enrichment (10 % cyclohexane and 20 % sodium chloride) method (Maruthiah et al. 2014, 2015).

### Enzyme assay and protein estimation

The protease assay was carried out by the method of Takami et al. (1989) using 1 % casein as a substrate. The amount of protease produced was measured with the help of a tyrosine standard graph. The protein content in all the samples was estimated using Bradford method. For this study readily used Bradford reagent was used (Sigma, USA).

### Effect of various marine wastes on protease production

In the investigation of suitable carbon source for protease production, growth was carried out in 250 ml Erlenmeyer flasks with 50 ml of basal medium containing 0.1 % K_2_HPO_4_ and 0.05 % MgSO_4_·7H_2_O (pH 9) and supplemented with 0.1–2 % (w/v) of marine wastes to be investigated such as SSP, CSP, SPP and SCSP (Shrimp and Crab shell powder at 1:1, 1:3 and 3:1 ratio, w/w) (Maruthiah et al. 2015).

### Production and purification of HOSP

In the investigation of suitable carbon source for protease production, growth was carried out in 500 ml Erlenmeyer flasks with 100 ml of basal medium containing Shrimp and Crab shell powder (3:1 w/w), 0.1 % K_2_HPO_4_ and 0.05 % MgSO_4_·7H_2_O, NaCl (100.00 g/l), calcium chloride 3.0 (g/l) were seeded with 5 % inoculum and incubated in shaking incubator (125 rpm) for 48 h. After incubation, the culture broth was centrifuged (4 °C at 12,000*g* for 20 min) and the crude enzyme was used for further purification.

### Purification of HOSP from *A. faecalis* APCMST-MKW6

The purification starts with 75 % ammonium sulphate precipitation and kept at 4 °C for 24 h. Ammonium sulphate fractions were resuspended in minimal volume of 50 mM Tris–HCl buffer (pH 7.2). The precipitates were collected through centrifugation at 6000*g* for 15 min and dissolved in 50 mM Tris–HCl buffer (pH 9.0) and dialysed against same buffer (4 °C) for 12 h. Then it was loaded on a DEAE-sepharose fast-flow column, pre-equilibrated with 50 mM Tris–HCl (pH 8.0). The unabsorbed protein fractions were eluted with the same buffer at a flow rate of 2 ml/min. The protease activity of individual eluted fractions was determined. Further the fractions showing the highest protease activity were pooled together and concentrated using ultra filtration unit (Amicon 10 kDa molecular weight cut-off device, Millipore, USA). The homogeneity and the molecular weight of the purified protease were determined by SDS-PAGE and further confirmed by zymogram analysis (Laemmli 1970; Garcia-Carreno et al. 1993).

### Biochemical properties of purified protease

The effect of different pH (4–10) and temperature (30–80 °C) on protease activity were studied. The effect of metal ions (MgCl_2_, CaCl_2_, ZnCl_2_, MnCl_2_, HgCl_2_, ZnSO_4_, MnSO_4_ and BaCl_2_ at 5 ppm), surfactants [poly ethylene glycol (PEG)], [sodium dodecyl sulphate (SDS)], triton × 100, Tween 20, 40, 60 and 80 at 5 mM), NaCl (5–25 %) inhibitor [phenyl methyl sulfonyl fluoride (PMSF), dithiothereitol (DTT)], iodoacetamide (IAA), β**-**mercaptoethanol and ethylene diamine tetra acetic acid (EDTA) at 5 mM], solvents (ethanol, methanol, hexane, acetone, DMSO, chloroform and benzene) and detergents (Ariel, Tide, Rin, Surf, Sunlight, Henko and Surf excel) were studied by following standard assay procedure (Maruthiah et al. 2014, 2015).

In the entire biochemical characterization studies, all the protease assays were performed, by the optimized temperature (60 °C) and pH (9.0). All experiments were conducted in triplicate and the results are represented as the mean values.

### Chitin extraction biological method

#### Biological method of deproteinization using crustacean shells

In the deproteinization experiment, the powdered shells from the crustaceans were individually mixed with above said halophilic production medium taken in flasks (solid to liquid ratio was 1:3 w/v) except casein acid hydrolysate, peptone and yeast extract. Then the flasks were sterilized at 121 °C and cooled. Further the candidate proteolyitc bacterial strain (*A. faecalis* APCMST-MKW6) was inoculated into the flasks. The flasks were incubated at 50 °C for 3 h. After incubation the reaction was stopped by heating the flasks for 20 min at 90 °C. Then, it was centrifuged at 5000 rpm for 20 min to separate soluble and insoluble fractions. The solid fraction was washed with distilled water and dried for 3 h at 60 °C. After that, the mineral contents (demineralization) were removed using the 2 N HCl for 24 h. Further, the samples were dried at 90 °C for 20 min. From the dried materials, the protein content was estimated using the standard protocol. Results were presented as means of experiments performed in triplicate (Yang et al. 2000).

### Antioxidant profile

#### DPPH radical scavenging activity

A volume of 500 µl each of experimental samples (culture supernatant, crude protease and commercial enzyme) was individually mixed with different concentrations (w/v) of (0.25–2.0 mg/ml) dried deproteinized shrimp shell powder (maximum deproteinized crustacean shell waste) with 99.5 % ethanol and 0.02 % DPPH in 99.5 % ethanol. Then the mixtures were incubated at 37 °C for 60 min in dark and the radical scavenging activity was measured at 517 nm. Distilled water was used as control. Lower absorption of the reaction mixture indicates higher DPPH radical scavenging activity (Maruthiah et al. 2015).

$${\text{Radical scavenging activity}}\;(\% ) = \frac{{A_{\text{control}} - A_{\text{sample}} }}{{A_{\text{control}} }} \times 100$$

where *A* is absorbance at 517 nm. The test was carried out in triplicate.

#### Reducing power assay

Each sample solution (1 ml) containing deproteinized (dried) shrimp shell waste at different concentrations of (0.25–2 mg/ml) was mixed with 2.5 ml of 0.2 M phosphate buffer (pH 6.6) and 2.5 ml of 1 % (w/v) potassium ferricyanide. The mixtures were then incubated for 30 min at 50 °C, followed by the addition of 2.5 ml of 10 % (w/v) trichloroacetic acid. The reaction mixtures were then centrifuged for 10 min at 10,000 rpm. Finally, 2.5 ml aliquot of the supernatant solution, from each sample mixture was mixed with 2.5 ml of distilled water and 0.5 ml of 0.1 % (w/v) ferric chloride. After 10 min incubation, the absorbance of the resulting solution was measured at 700 nm. Higher absorbance of the reaction mixture indicated maximum reducing power. Values presented are the mean of triplicate analyses (Maruthiah et al. 2015).

#### Metal chelating measurement

A known volume (1.1 ml) of diluted sample was mixed with 100 µl of FeCl2. The reaction was initiated by the addition of 400 µl of ferrozine, and after 10 min when the mixture reached equilibrium, the absorbance was read at 562 nm. A control was maintained in the same manner, and here distilled water was used instead of sample (Maruthiah et al. 2015). The percentage of inhibition of the complex ferrozine-Fe^2+^ was calculated using the following equation:

$${\text{Inhibition}}\; ( {\text{\%}})\,=\,\frac{{A_{\text{control}} \; - \;A_{\text{sample}} }}{{A_{\text{control}} }} \times 100$$

where *A* is absorbance at 562 nm. Results were carried out in triplicate.

#### Stain removal

The application of protease as a detergent additive was studied on white cotton cloth pieces (5 cm × 5 cm) stained with human blood and natural colorants like green leaves, tea, coffee, chocolate, tomato, pomegranate, Greece and beetroot. After incubation at 60 °C for 30 min, the cloth pieces were taken out, rinsed with distilled water, and dried. Visual examination of various pieces exhibited the effect of enzyme in the removal of stains. Untreated cloth pieces were taken as control (Maruthiah et al. 2015).

## Results and discussion

### Microorganism

The HOSP producing strain APCMST-MKW6 was isolated from marine water of Manakudi coast, Tamilnadu, India and screened for their protease activity. The morphological and biochemical, 16S rRNA gene sequence analysis were confirmed the identity of the strain MKW6 as *A. faecalis* APCMST-MKW6 (GenBank Accession No. KF009687). Similar to this study, limited number of reports were available for the isolation of halophilic organic solvent tolerant proteolytic bacteria from the costal sediment and sea water samples (Shah et al. 2010; Karan and Khare 2011; Sinha and Khare 2012; Raut et al. 2013; Annamalai et al. 2012, 2013, 2014a, b; Maruthiah et al. 2014, 2015).

### Effect of various marine wastes on protease production

**Fig. 1:**
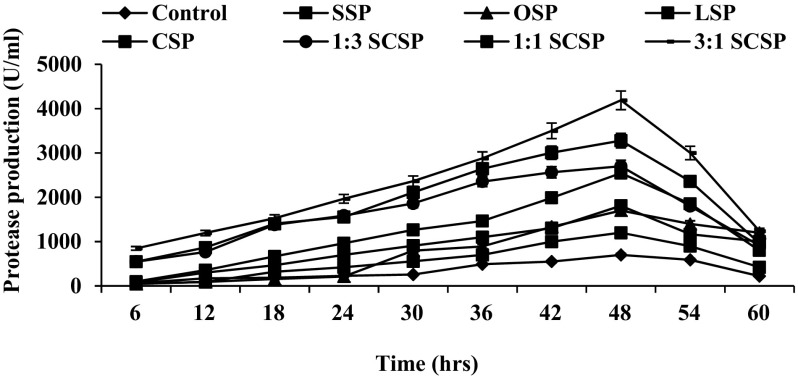
Effect of protease production using marine crustacean shell waste. *Each value* represents the mean of three experiments and the *error bars* indicate ±SD

**Fig. 2:**
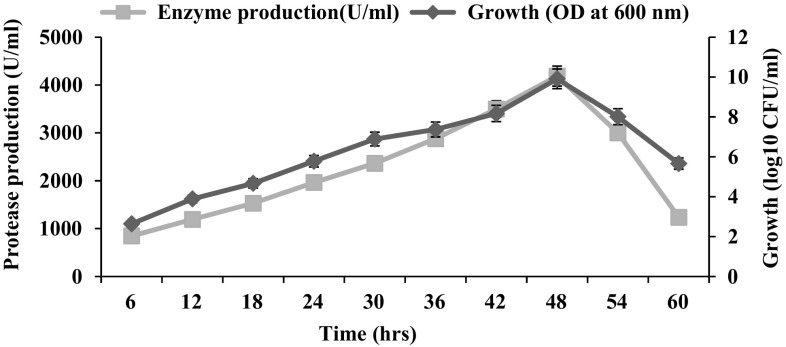
Kinetics of bacterial cell growth and production of HOSP by optimized medium. *Each value* represents the mean of three experiments and the *error bars* indicate ±SD

The effect of various crustacean shell wastes on protease production was carried out in basal medium at 1 % concentration (3:1 w/v). Here, maximum protease production was noticed in SSP (1987.74 U/ml) and CSP (1808.41 U/ml) added medium. Hence, further study was performed with different ratios of shrimp and crab shell powder (SCSP). The results indicated that the protease production was maximum (4186.65 U/ml) at 3:1 % ratio of SCSP during 48 h of incubation and it was low in 1:1 % SCSP (3277.11 U/ml) and 1:3 % SCSP (2700.55 IU/ml) (Figs. 1, 2). In consistence with the present study, Annamalai et al. (2012, 2014a, b) and Maruthiah et al. (2014, 2015) have reported enhanced protease production by *Bacillus halodurans* CAS6 (3413 U/ml), *Bacillus firmus* CAS7 (2289 U/ml), *Bacillus alveayuensis* CAS 5 (2675 U/ml) and *Bacillus* sp. APCMST-RS7 (139 U/ml) when SCSP was used as a substrate.

### Purification of HOSP by *A. faecalis* APCMST-MKW6

**Fig. 3:**
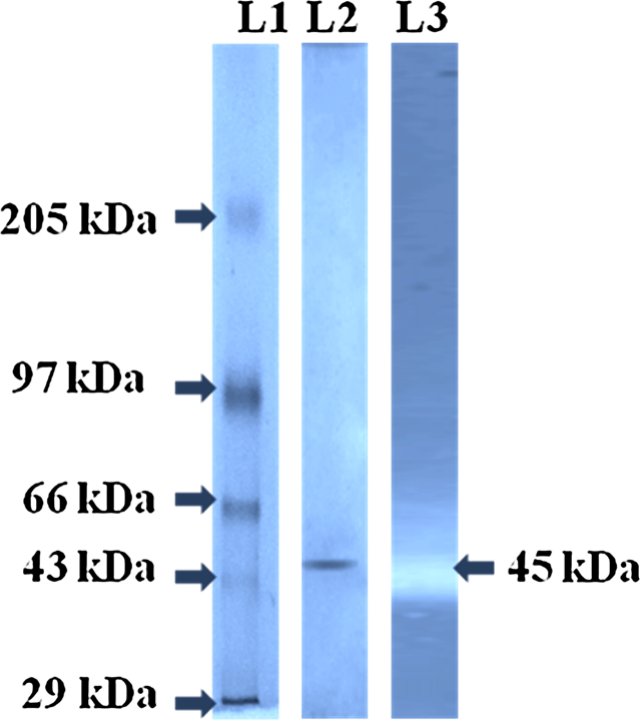
SDS-PAGE analysis of purified HOSP. *Lane 1*—molecular weight markers. *Lane 2*—purified protease. *Lane 3*—zymography

The HOSP from *A. faecalis* APCMST-MKW6 was purified to the homogeneity by a combination of ammonium sulphate precipitation, dialysis, ultra filtration and ion-exchange chromatography. After final purification, the protease was purified with 16.39 fold purity and the overall yield registered was 21.67 %. The specific activity of the purified enzyme was 70.34 U/mg (Table 1). Earlier, the purification and characterization of halophilic organic solvent tolerant protease from various marine coastal proteolytic bacterial species such as; *B. cereus* (Shah et al. 2010), *Virgibacillus* sp. EMB13 (Sinha and Khare 2012), *B. halodurans* CAS6 (Annamalai et al. 2013), *B. firmus* CAS 7 (Annamalai et al. 2014a, b), *B. flexus* APCMST-RS2 (Maruthiah et al. 2014), *Bacillus* sp. APCMST-RS7 (Maruthiah et al. 2015). In this study, the molecular weight of the purified protease was confirmed as 45 kDa through SDS-PAGE and zymogram analysis (Fig. 3). Related molecular weight proteases with comparable specific activity and purity were reported from several marine aquatic halophilic organic solvent tolerant proteolytic strains such as *B. flexus* APCMST-RS2 (44.3 kDa), *B. alveayuensis* CAS5 (33 kDa), and *Bacillus* sp. APCMST-RS7 (32 kDa) (Annamalai et al. 2014b; Maruthiah et al. 2014, 2015).

**Table 1:** Summary of purification of protease produced by *Alcaligenes faecalis* APCMST-MKW6

Purification steps	Total activity (U/mL)	Total protein (mg)	Specific activity (U/mg)	Yield (%)	Purification fold
Culture filtrate	4244.19 ± 2.58	988.14 ± 1.08	4.29 ± 0.28	100 ± 0.00	1 ± 0.00
Ammonium sulphate precipitation	2189.78 ± 1.17	185.19 ± 0.87	11.82 ± 0.59	51.59 ± 1.27	2.75 ± 0.49
DEAE sepharose	920.10 ± 0.98	13.08 ± 0.58	70.34 ± 2.27	21.67 ± 0.60	16.39 ± 0.19

All the results were presented as the mean ± SD

### Characterization

**Fig. 4:**
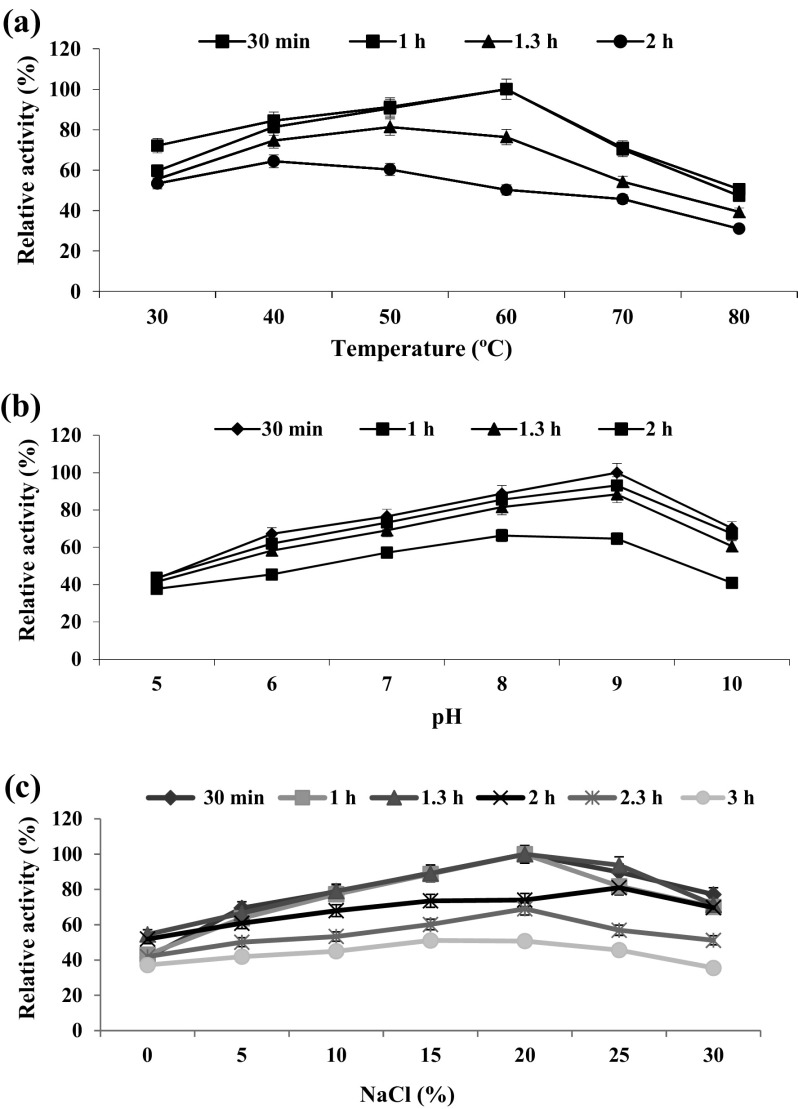
Effect of physical factors on protease activity. **a** Effect of temperature on protease activity. **b** Effect of pH on protease activity. **c** Effect of NaCl on protease activity. *Each value* represents the mean of three experiments and the *error bars* indicate ±SD)

In this study, the effect of optimum temperature, pH and NaCl inferred that the HOSP showed maximum activity at 60 °C, pH 9.0 and 20 % NaCl after 1.30 h of incubation time (Fig. 4a, b, c). Hence this protease was classified as a moderately thermoactive alkaline halophilic protease. In accordance with this study, similar kind of halophilic organic solvent tolerant proteases were previously reported from different marine bacterial species such as *B. firmus* CAS7 and *B. alveayuensis* CAS5 (Annamalai et al. 2014a, b). The influence of different chemicals on protease activity was also screened in this study. Among the tested chemicals MnCl_2_, casein, Ariel and hexane were enhanced the protease activity to the maximum level. Further, MgCl_2_, CaCl_2_, and BaCl_2_ (metal ions); SDS (surfactant); Rin and Tide (detergents) and isopropanol, acetonitrile, methanol, *N*-butanol and ethanol (solvents) were also favored the protease activity by candidate bacterium. In correlation with this study enhanced protease activity was also reported from different halophilic organic solvent tolerant *Bacillus* spp. Of these tested additives (Annamalai et al. 2014a, b; Maruthiah et al. 2014, 2015). Further proteases are classified based on their sensitivity to various inhibitors. In this study, the protease activity was effectively inhibited by serine (PMSF) and metalloprotease (EDTA) inhibitors and thus it evidenced that this enzyme is serine metalloprotease (Tables 2, 3, 4). This result suggested the presence of serine residue near the active site of the metalloprotease. Earlier reports also confirmed that β-mercaptoethanol and DTT stimulate the enzyme activity, suggesting that both are thiol-dependent serine proteases (Annamalai et al. 2014a, b; Maruthiah et al. 2014, 2015).

**Table 2:** Effect of different metal ions and surfactants on protease activity

Metal ions	Relative activity (%)	Surfactants	Relative activity (%)
Barium chloride	119.00 ± 1.53	Tween 20	82.19 ± 1.25
Calcium chloride	133.47 ± 1.66	Tween 40	91.24 ± 1.41
Magnesium chloride	141.12 ± 0.85	Tween 80	153.47 ± 1.89
Manganese chloride	159.22 ± 2.42	Triton × 100	79.17 ± 0.67
Mercuric chloride	12.09 ± 0.18	PEG	34.47 ± 0.91
Zinc chloride	15.50 ± 0.25	SDS	118.19 ± 1.67
Copper sulphate	81.70 ± 0.74	control	100.00 ± 0.00
Control	100.00 ± 0.00		

*PEG* poly ethylene glycol, *SDS* sodium dodecyl sulphate

All the results were presented as the mean ± SD

**Table 3:** Effect of different inhibitors, substrate and commercial detergents on protease activity

Inhibitors	Relative activity (%)
PMSF	22.60 ± 0.25
EDTA	39.57 ± 0.81
DTT	144.11 ± 1.34
Iodoacetamide	169.43 ± 1.55
β-mercaptoethanol	154.17 ± 1.39
Control	100 ± 0.00
Substrates	
Casein	100 ± 0.00
BSA	31.0 ± 0.39
Gelatin	58.80 ± 0.91
Commercial detergents	
Surf excel	102.17 ± 2.09
Ariel	163.10 ± 2.16
Tide	119.98 ± 1.98
Rin	133.00 ± 2.09
Sunlight	117.26 ± 2.01
Control	100 ± 0.00

All the results were presented as the mean ± SD

**Table 4:** Effect of different organic solvents at two different concentrations (10 and 20 %) on protease activity

Organic solvents	Relative activity (%)	
	10	20
Isopropanol	152.71 ± 1.58	140.20 ± 1.20
Acetonitrile	148 ± 1.69	139 ± 1.68
Hexane	174 ± 1.58	161 ± 1.36
Benzene	79 ± 0.75	84 ± 0.92
*N*-butanol	111 ± 1.04	102 ± 1.01
Ethanol	107 ± 0.97	119 ± 1.09
Methanol	131 ± 1.32	144.17 ± 1.60

All the results were presented as the mean ± SD

### Application studies

#### Stain removal in detergent industry

**Fig. 5:**
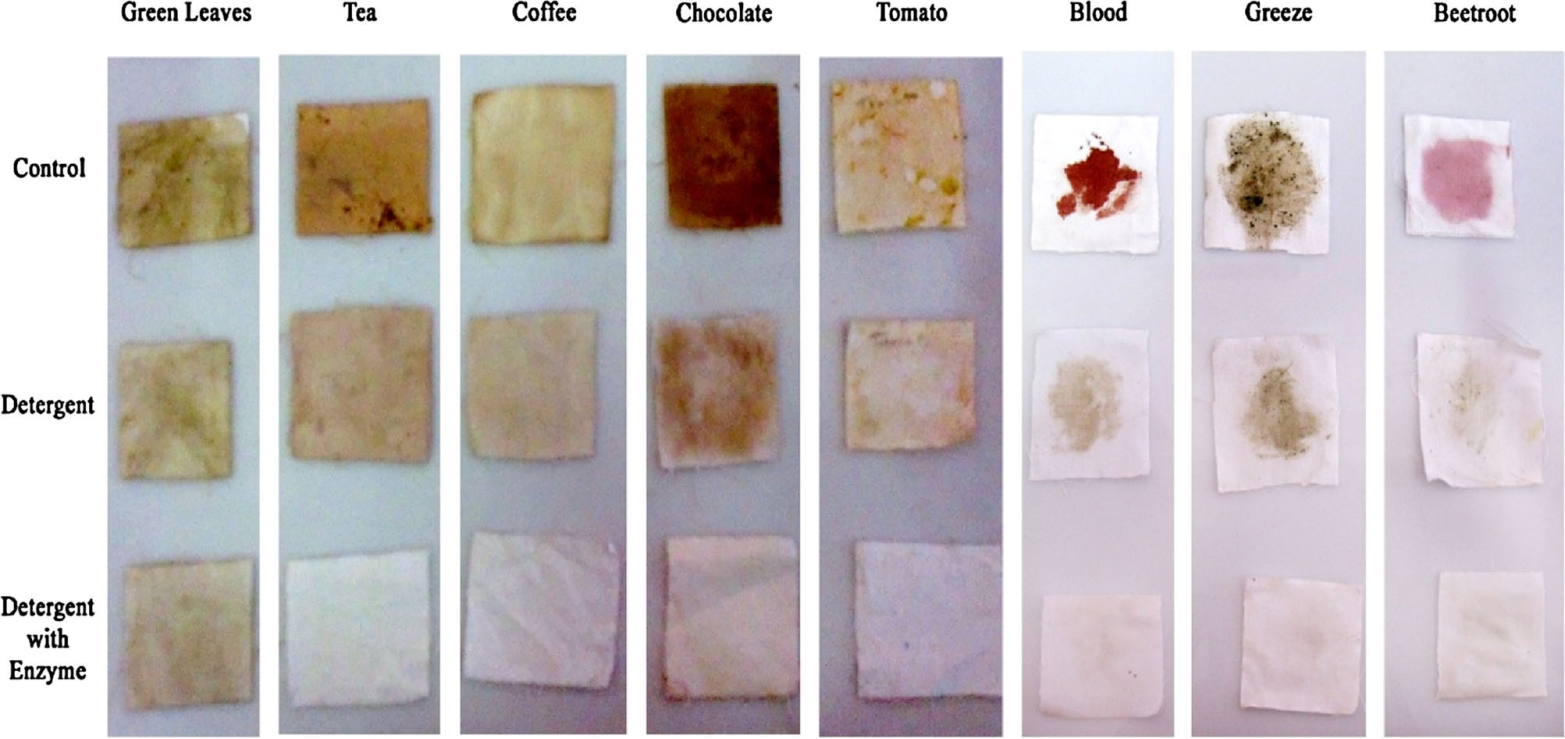
Stain removal activity of the crude HOSP from *Alcaligenes faecalis* APCMST-MKW6. Detergent (Ariel)—7 mg/ml, crude enzyme from APCMST-MKW6—4244 U/ml

In this study, the protease from *A. faecalis* APCMST-MKW6 showed better stain removal when applied with aqueous solution (Fig. 5). In agreement with this report, the halophilic proteases from various *Bacillus* species such as *B. subtilis* (Jellouli et al. 2011), *B. halodurans* CAS6 (Annamalai et al. 2012) and *Bacillus* sp. APCMST-RS7 (Maruthiah et al. 2015) also showed better efficient stain removal ability. The results explored the possibility of using the HOSP from *A. faecalis* APCMST-MKW6 in detergent industry.

### Marine fish waste based applications

#### Deproteinization by *A. faecalis* APCMST-MKW6

**Fig. 6:**
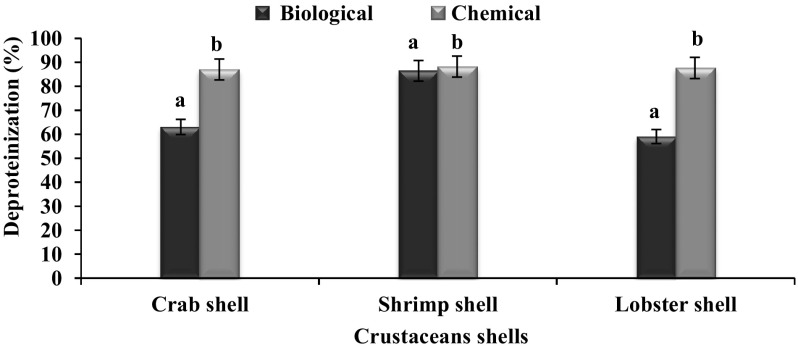
Effect of deproteinization on different crustacean shells. *Each value* represents the mean of three experiments and the *error bars* indicate ±SD. *Bars* having different alphabets in the respective crustacean shell wastes are statistically significant (*P* < 0.05)

Deproteinization is one of the most important industrial applications which was successfully carried out in this study using chemical and biological approaches. Suggesting the efficiency of the bio-deproteinization process, the culture supernatant from *A. faecalis* APCMST-MKW6 was effectively deproteinized the shrimp shell to the maximum of 86.46 % within 7 days of fermentation, followed by crab (63.08 %) and lobster shells (59.07 %), respectively (Fig. 6). Further it inferred that, the biological deproteinization of shrimp shell was comparable with chemical method, and it was not statistically significant (*P* > 0.05). This results find support from the earlier findings that, the different proteolytic bacterial isolates were effectively deproteinized the crustacean shell wastes in the preparation of chitin (Yang et al. 2000; Jellouli et al. 2011; Ghorbel-Bellaaj et al. 2012; Maruthiah et al. 2015; Hajji et al. 2015). The reclamation processing of crustacean shell wastes by bioconversion emerged as an alternative solution to reduce the environmental problems associated with crustacean processing industries.

#### Antioxiant activity (DPPH activity, reducing power and metal chelating activity)

**Fig. 7:**
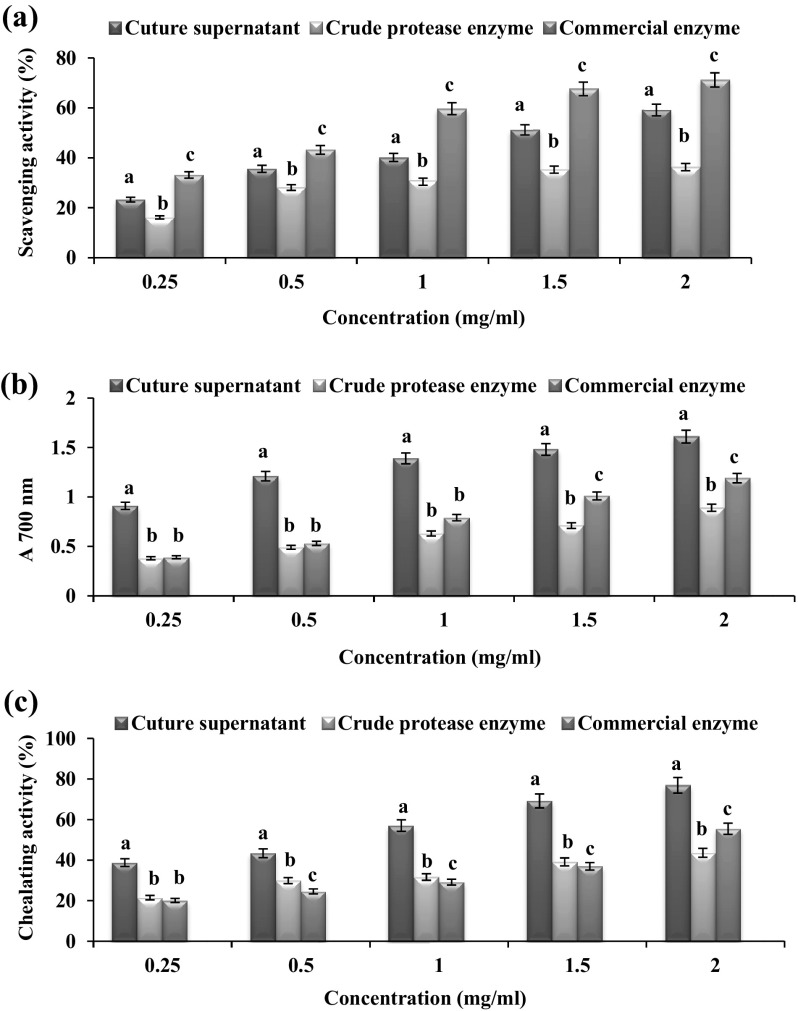
Antioxidant activity of crustacean shell waste. **a** Effect of DPPH scavenging activity by different concentrations of shrimp shell substrate. **b** Reducing power. **c** Metal chelating activity. *Each value* represents the mean of three experiments and the *error bars* indicate ±SD. *Bars* having different alphabets at the respective concentrations are statistically significant (*P* < 0.05)

The antioxidant profile of the shrimp shell hydrolasate with culture supernatant, crude protease (HOSP) and commercial protease was determined. The result inferred that the maximum DPPH radical scavenging activity (71.16 %), reducing power (OD = 1.63) and metal chelating activities (79.64 %) were recorded at 2 mg/ml concentration using shrimp shell as a substrate along with candidate marine proteolytic bacterial culture supernatant compared to the tested commercial (Alcalase) and crude protease. (Figure 7a, b, c). The DPPH radical scavenging assay is a widely used method for evaluating the ability to scavenge free radicals generated from DPPH reagent. DPPH is a stable free radical, which can be reduced by a proton donating substrate such as an antioxidant, causing the decolorization of DPPH and the reduction of the absorbance at 517 nm. The decrease in absorbance is taken as a measure for radical scavenging activity. In consistence with this study, Annamalai et al. (2012) reported the alkaline protease from B. halodurans CAS6 had attained 96 % maximum antioxidant activity using shrimp shell as a substrate. Similarly, Ghorbel-Bellaaj et al. (2012) examined the hydrolysate obtained with strain A1 (*B. pumilus*) was found to be the most active radical-scavenger (IC50 = 0.3 mg/ml) followed by hydrolysates obtained by strain An6 (*Bacillus amyloliquefaciens*), strain RP1 (*Bacillus licheniformis*), strain A26 (*Bacillus subtilis*) and strain A21 (*Bacillus mojavencis*), which showed comparable IC50 ranging from 0.5 to 0.6 mg/ml. The highest IC50 (1.4 mg/ml) was obtained with strain SV1 (*B. cereus*) hydrolysate. Maruthiah et al. (2015) observed that the shrimp shell hyrolasate obtained from *Bacillus* sp. APCMST-RS7 showed 80 % maximum DPPH radical-scavenging activity. The obtained results inferred that shrimp waste hydrolysates probably contained peptides or chitooligosaccharides, which were electron donors and could react with free radicals to convert them to more stable products and terminate the radical chain reaction (Manni et al. 2010; Ghorbel-Bellaaj et al. 2012). Thus, the fermented shrimp shell waste liquor has provided a complex mixture of compounds able to quench DPPH.

The reducing power assay could be used to evaluate the ability of antioxidant to transfer electron or hydrogen (Yildirim et al. 2003). Similar to this study, Ghorbel-Bellaaj et al. (2012) examined the reducing power of tested hydrolysates (strain RP1, A26, A21, An6 and SV1) increased with increasing concentrations (0.25–2 mg/ml) along with the culture supernatant of strain A1 (*B. pumilus*). The highest activity (DO 700 nm = 1.55 at 1.5 mg/ml) was found in hydrolysate produced by A1 bacterial strain. Recently, the maximum reducing power activity (DO 700 nm = 1.71 at 2.0 mg/ml) in shrimp shell hydrolysate produced by culture supernatant from *Bacillus* sp. APCMST-RS7 was also reported (Maruthiah et al. 2015). The reducing power could mainly be attributed to the bioactive compounds associated with antioxidant activity (He et al. 2000). These bioactive compounds might be present in the shrimp waste fermented supernatant, including phenolics, oligopeptides, or chitooligosaccharides; they are good electron donors and can terminate the radical chain reactions by converting free radicals to more stable products (Ghorbel-Bellaaj et al. 2012).

Metal chelation can result in prevention of metal redox cycling, occupation of all metal coordination sites, formation of insoluble metal complex, steric hindrance of interaction between metals, and formation of lipid intermediates (Hider et al. 2001; Moridani et al. 2003). Similar to this study, shrimp shell hydrolysate obtained with *B. licheniformis* RP1 exhibited the highest (98 % at 5 mg/ml) ferrous chelating activity (Ghorbel-Bellaaj et al. 2012). Similarly, our previous study also reported the maximum chelating activity (98 % at 5 mg/ml) obtained with shrimp shell hydrolasate with *Bacillus* sp. APCMST-RS7. Because ferrous ions are the most effective pro oxidants, the highest chelating ability of the APCMST-RS7 culture supernatant would be beneficial (Maruthiah et al. 2015). The difference between DPPH scavenging, reducing power and ferrous ion chelating abilities might be related to the different antioxidant compounds present in the culture supernatants. Still the exact mechanism of radical scavenging activity is not clear, it is attributed to amino and hydroxyl groups reacting with unstable free radicals, which facilitate formation of stable macromolecule radicals.

## Conclusion

Low cost protease production, through microbial reclamation of marine wastes seems to be a judicious approach because of its eco-friendly nature. The production of inexpensive proteinolytic enzymes not only solves environmental problems, but also promotes the economic value and utilization of marine wastes. The remarkable properties of halophilic organic solvent tolerant protease produced by *A. faecalis* APCMST-MKW6 such as temperature, pH and NaCl tolerance and also compatibility with surfactants, metals will make this enzyme as a potential product for the development of sustainable industrial process for waste management. Also, deproteinization and antioxidants properties of shrimp shell hydrolasate showed further industrial applications of the HOSP of APCMST-MKW6.

## References

[CR1] Annamalai N, Rajeswari MV, Thavasi R, Vijayalakshmi S, Balasubramanian T (2012). Optimization, purification and characterization of novel thermostable, haloalkaline, solvent stable protease from *Bacillus halodurans* CAS6 using marine shellfish wastes: a potential additive for detergent and antioxidant synthesis. Bioprocess Biosyst Eng.

[CR2] Annamalai N, Rajeswari MV, Thavasi R, Vijayalakshmi S, Balasubramanian T (2013). Optimization, purification and characterization of novel thermostable, haloalkaline, solvent stable protease from *Bacillus halodurans* CAS6 using marine shellfish wastes: a potential additive for detergent and antioxidant synthesis. Bioprocess Biosyst Eng.

[CR3] Annamalai N, Rajeshwari MV, Sahu SK, Balasubramanian T (2014). Extraction, purification and application of thermostable and halostable alkaline protease from *Bacillus alveayuensis* CAS 5 using marine wastes. Food Bioprod Process.

[CR4] Annamalai N, Rajeshwari MV, Sahu SK, Balasubramanian T (2014). Purification and characterization of solvent stable, alkaline protease from *Bacillus firmus* CAS7 by microbial conversion of marine wastes and molecular mechanism underlying solvent stability. Process Biochem.

[CR5] Doukyu N, Ogino H (2010). Organic solvent-tolerant enzymes. Biochem Eng J.

[CR6] Garcia-Carreno FC, Dimes CE, Haard NF (1993). Substrate gel electrophoresis for composition and molecular weight of proteinases of proteinaceous proteinase inhibitor. Anal Biochem.

[CR7] Ghorbel-Bellaaj O, Hana Maalej IY, Hajji S, Nasri M (2012). Chitin extraction from shrimp shell waste using *Bacillus* bacteria. Int J Biol Macromol.

[CR9] Haddar A, Hmidet N, Ghorbel-Bellaaj O, Fakhfakh-Zouari N, Sellami-Kamoun A, Nasri M (2011). Alkaline proteases produced by *Bacillus licheniformis* RP1 grown on shrimp wastes: application in chitin extraction, chicken feather-degradation and as a dehairing agent. Biotechnol Bioche Eng.

[CR10] Hajji S, Ghorbel-Bellaaj O, Younes I, Jellouli K, Nasri M (2015). Chitin extraction from crab shells by bacillus bacteria. Biological activities of fermented crab supernatants. Int J Biol Macromol.

[CR11] He ZD, Lau KM, Xu HX, Li PC, But PPH (2000). Antioxidant activity of phenylethanoid glycosides from *Brandisia hancei*. J Ethnopharmacol.

[CR12] Hider RC, Liu ZD, Khodr HH (2001). Metal chelation of polyphenols. Methods Enzymol.

[CR13] Islam MS, Khan S, Tanaka M (2004). Waste loading in shrimp and fish processing effluents: potential source of hazards to the coastal and nearshore environments. Mar Pollut Bull.

[CR14] Jellouli K, Ghorbel-Bellaaj O, Ayed HB, Manni L, Agrebi R, Nasri M (2011). Alkaline-protease from *Bacillus licheniformis* MP1: purification, characterization and potential application as a detergent additive and for shrimp waste deproteinization. Process Biochem.

[CR15] Karan R, Khare SK (2011). Stability of haloalkaliphilic *Geomicrobium* sp. protease modulated by salt. Biochemistry.

[CR16] Laemmli DK (1970). Cleavage of structural proteins during the assembly of the head of bacteriophage T4. Nature.

[CR17] Manni L, Jellouli K, Ghorbel-Bellaaj O (2010). An oxidant- and solvent-stable protease produced by *Bacillus cereus* SV1: application in the deproteinization of shrimp wastes and as a laundry detergent additive. Appl Biochem Biotechnol.

[CR18] Maruthiah T, Esakkiraj P, Immanuel G, Palavesam A (2014). Alkaline serine protease from *Bacillus fluxus* AP-CMST-RS2P: purification and characterization. Curr Biotechnol.

[CR19] Maruthiah T, Immanuel G, Palavesam A (2015). Purification and characterization of halophilic organic Solvent tolerant protease from marine *Bacillus* sp. APCMST-RS7 and its antioxidant potentials. Proc Natl Acad Sci India Sect B Biol.

[CR20] Moridani MY, Pourahmad J, Bui H, Siraki A, Brien OPJ (2003). Dietary flavonoid iron complexes as cytoprotective superoxide radical scavengers. Free Rad Biol Med.

[CR21] Rahman RNZRA, Geok LP, Basri M, Salleh AB (2006). An organic solvent-stable alkaline protease from *Pseudomonas aeruginosa* strain K: enzyme purification and characterization. Enzyme Microb Technol.

[CR22] Rajkumar R, Jayappriyan KR, Rengasamy R (2011). Purification and characterization of a protease produced by *Bacillus megaterium* RRM2: application in detergent and dehairing industries. J Basic Microbiol.

[CR23] Raut GR, Chakraborthy S, Chopade BA, Kolkare CR (2013). Isolation and characterization of organic solvent tolerant protease from alkaliphilic marine *Saccharopolyspora* species. Ind J Biotechnol.

[CR24] Sachindra NM, Bhaskar N, Mahendrakar NS (2005). Carotenoids in different body components of Indian shrimps. J Sci Food Agric.

[CR25] Shah K, Mody K, Keshri J, Jha B (2010). Purification and characterization of a solvent, detergent and oxidizing agent tolerant protease from *Bacillus cereus* isolated from the Gulf of Khambhat. J Mol Catal B Enzym.

[CR26] Sinha R, Khare SK (2012). Isolation of a *Virgibacillus* sp. EMB13: characterization of its protease for detergent application. Indian J Biotechnol.

[CR27] Takami H, Akiba T, Horikaoshi K (1989). Production of extremely thermostable alkaline protease from *Bacillus* Sp. No. AH-101. Appl Microbiol Biotechnol.

[CR28] Yang JK, Shih IL, Tzeng YM, Wang SL (2000). Production and purification of protease from a *Bacillus subtilis* that can deproteinize crustacean wastes. Enzyme Microb Technol.

[CR29] Yildirim M, Baysal V, Serhat Inaloz H, Kesici D, Delibas N (2003). the role of oxidants and antioxidants in generalized vitiligo. J Dermatol.

